# Quality of Social Media and Web-Based Information Regarding Inappropriate Nuclear Cardiac Stress Testing and the Choosing Wisely Campaign: A Cross-Sectional Study

**DOI:** 10.2196/ijmr.7210

**Published:** 2017-05-04

**Authors:** David E Winchester, Diana Baxter, Merry J Markham, Rebecca J Beyth

**Affiliations:** ^1^ Cardiology Section Medical Service Malcom Randall VA Medical Center Gainesville, FL United States; ^2^ Division of Cardiovascular Medicine Department of Medicine University of Florida Gainesville, FL United States; ^3^ College of Medicine University of Florida Gainesville, FL United States; ^4^ Division of Hematology & Oncology Department of Medicine University of Florida Gainesville, FL United States; ^5^ Department of Medicine College of Medicine University of Florida Gainesville, FL United States; ^6^ Geriatric Research Education and Clinical Centers Malcom Randall VA Medical Center Gainesville, FL United States

**Keywords:** myocardial perfusion imaging, health services research, Internet, unnecessary procedures

## Abstract

**Background:**

The World Wide Web and social media provide the public with access to medical information unlike any other time in human history. However, the quality of content related to cardiac stress testing is not well understood.

**Objective:**

The aim of our study was to evaluate the quality of content on the Internet relating to the use of cardiac nuclear stress testing and the Choosing Wisely campaign.

**Methods:**

We searched the World Wide Web, Google Video (including YouTube), and Twitter for information relating to these two topics. Searches were performed using English language terms from a computer in the United States not logged into any personal user accounts. Search results were reviewed for discussion of specific topics including radiation risk, accuracy of testing, alternative testing options, and discouragement of inappropriate test use.

**Results:**

We evaluated a total of 348 items of content from our searches. Relevant search results for Choosing Wisely were fewer than for other search terms (45 vs 303). We did not find any content which encouraged inappropriate testing (ie, screening in low risk individuals or testing prior to low risk operations). Content related to Choosing Wisely was more likely to discourage inappropriate testing than search results for other terms (29/45, 64% vs 12/303, 4.0%, odds ratio 43.95, 95% CI 17.6-112.2, *P*<.001).

**Conclusions:**

The Internet content on nuclear stress tests consistently discouraged inappropriate testing. The Choosing Wisely content was more likely to discourage inappropriate testing, less relevant content was available. Generating authoritative content on the Internet relating to judicious use of medical interventions may be an important role for the Choosing Wisely campaign.

## Introduction

Patients are increasingly using the Internet and social media to understand health conditions and for decisions about proposed medical interventions. Increasing evidence suggests that the Internet and social media are effective at driving health behaviors [1]. Misinformation and patient demand may contribute to the estimated US $200 billion in unnecessary medical services within the US healthcare system [2]. In an effort to combat this issue, the American Board of Internal Medicine Foundation and numerous other medical organizations have partnered in the Choosing Wisely campaign, a movement to raise awareness among physicians and patients about unnecessary tests, procedures, and treatments. The program aims to help patients “choose care that is supported by evidence, not duplicative of other tests, free from harm, and truly necessary.” Inappropriate use of myocardial perfusion imaging (MPI) is discouraged on multiple Choosing Wisely lists, especially when applied in asymptomatic and low risk patient populations.

Despite efforts such as the Choosing Wisely campaign to better inform both patients and doctors about low-value care [3], inappropriate nuclear MPI are still commonly performed [4]. Given the semielective and outpatient nature of many MPI, patients could conceivably use the Internet to obtain information about the test before having the MPI performed.

We conducted this investigation to evaluate the quality and quantity of publicly available information on the Internet and social media regarding nuclear MPI. We specifically sought evidence of misinformation on MPI that could contribute to inappropriate MPI. We hypothesized that content related to the Choosing Wisely campaign would be more likely to contain information related to the appropriateness of testing than general Internet content on MPI.

## Methods

We conducted a descriptive cohort study using searches of the World Wide Web using Google Web Search (Mountain View, CA), video clips using Google Video Search (Mountain View, CA), and social media content on Twitter (San Francisco, CA). We did not include other platforms, such as Facebook, where search results are based on the user’s personal contact group and do not provide an open public-facing search. Three search terms were used on each platform: “nuclear stress test,” “myocardial perfusion imaging,” and “Choosing Wisely stress test.” The only exclusion criteria were irrelevance (not mentioning nuclear stress tests specifically) and non-English language. We did not use any advanced search features or apply “hashtags” in conducting the searches. The searches were performed from a computer located in the United States and none were accessed while logged into a private account in order to minimize any bias in the results provided by each search engine.

Data were collected from June 2015 to August 2015 by DB. Search results were stored in a custom, secure, Web-based database, Research Electronic Data Capture or REDCap [5]. Each relevant search result was categorized by the source (Web, video, or Twitter) and the author type: patient, physician, hospital or practice, academic, news or informational, or other. The specific data elements gathered for each piece of content were the presence of any discussion on: (1) radiation risk of nuclear stress testing, (2) alternative testing options, (3) the accuracy of MPI for detecting heart disease, and (4) discouragement of inappropriate testing. Sampling in each search was continued until further search results were considered futile.

The primary outcome of interest was to compare how frequently the topic of inappropriate MPI was mentioned based on the search result employed. Secondary outcomes were to report descriptive characteristics of the search results including the author type and distribution across different Internet and social media platforms. As a descriptive study, no formal power calculation was performed a priori. The research protocol was reviewed by our institutional review board and classified as exempt from further review. The study design had no direct human involvement. No changes to the study design, conduct, or outcomes were made after initiation. Selected pairwise comparisons were made using Fisher exact and chi-square tests using SPSS version 21 (IBM, Armonk NY). *P*<.05 was considered significant.

## Results

**Figure 1:**
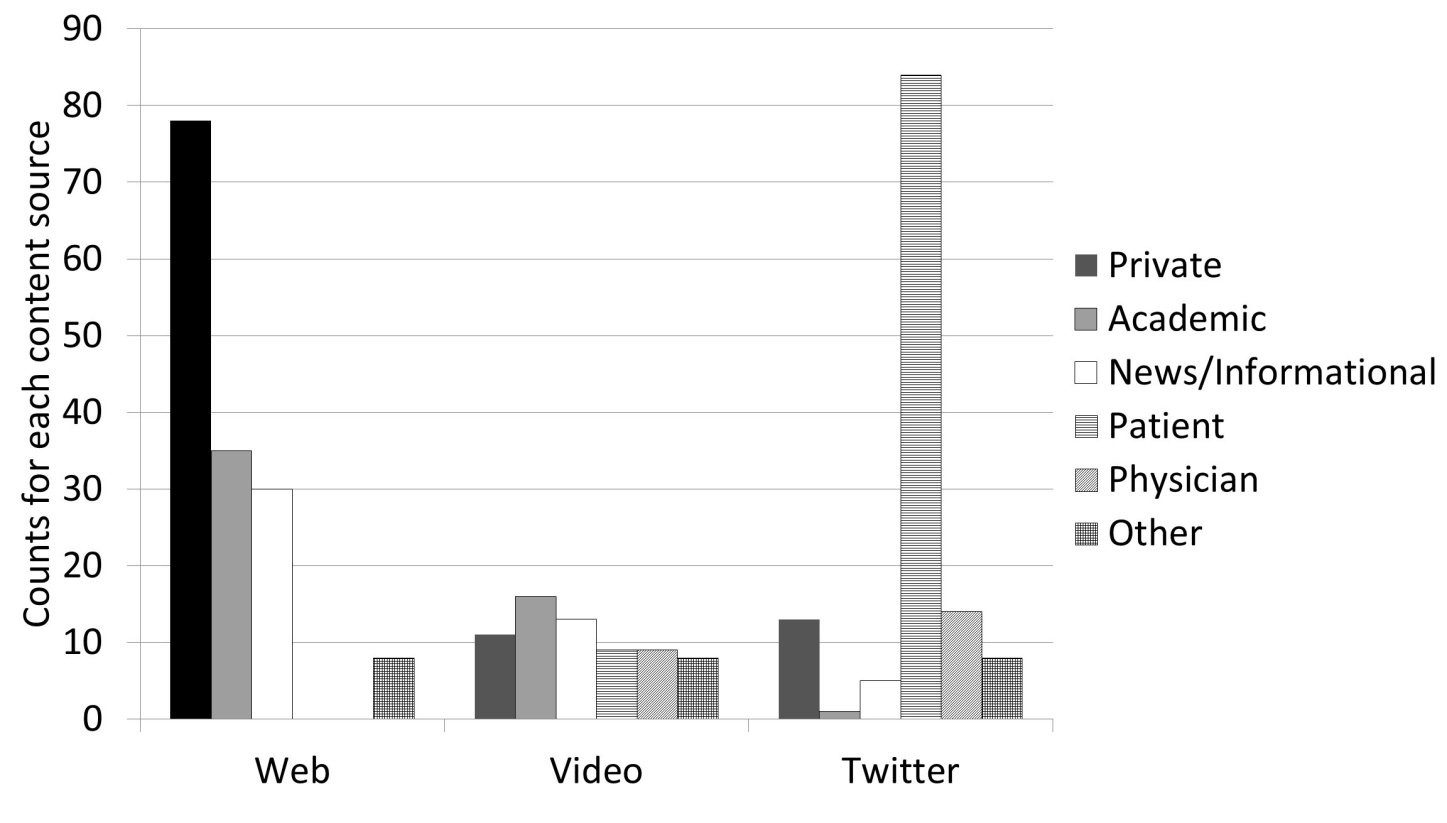
The distribution of content author types differed across the platforms we analyzed. Web content came primarily from private and academic practices, while Twitter content was primarily from patients, and video content was authored by a variety of sources.

A total of 456 search results were analyzed with 348 retained after 108 were excluded as duplicative, irrelevant, or non-English language. The plurality of relevant results came from the Web (n=154) followed by Twitter (n=125) and then video sources (n=69). The author type was different for each source; whereas video content was seen from all author types, Web results were predominantly from private and academic practices (113/ 154, 73.3%) and the Twitter search yielded mostly results from patient authors (84 /125, 67.2%). Content from individual physicians on the three platforms was minimal (23 /347, 6.6% overall; Figure 1).

The content of relevant search result material differed based on the search term used (see Table 1).

Searching for Choosing Wisely yielded the fewest results of the 3 search terms (Choosing Wisely n=45, nuclear stress n=223, MPI n=80). Of note, none of the search results actively encouraged inappropriate MPI (such as for screening in asymptomatic patients, annual testing in heart disease patients, or routine use prior to invasive procedures or operations). Results of the “Choosing Wisely” search were more likely to discourage inappropriate MPI than results for “myocardial perfusion imaging” or “nuclear stress test” (n=29 of 45 vs 12 of 303, odds ration [OR] 44.0, 95% CI 17.6-112.2, *P*<.001). “Choosing Wisely” results were also more likely to discuss the accuracy of MPI (20 of 45 vs 15 of 303, OR 15.4, 95% CI 6.6-36.3) or radiation risks (18 of 45 vs 64 of 303, OR 2.5, 95% CI 1.2-5.0, *P*=.005). Discussion of alternative testing options did not differ between the search terms (3 of 45 vs 20 of 303, OR 1.0, 95% CI 0.2-3.8, *P*>.99).

**Table 1:** Search results for n=348 items of content on the Internet or social media.

Search term	Source	Radiation n (%)	Alternatives n (%)	Accuracy n (%)	Inappropriate use discouraged n (%)
Nuclear stress test	Web (n=92)	17 (18.4)	4 (4.3)	4 (4.3)	1 (1.1)
	Video (n=43)	12 (27.9)	5 (11.6)	3 (7.0)	2 (4.7)
	Twitter (n=88)	13 (14.7)	1 (1.1)	0 (0.0)	1 (1.1)
Myocardial perfusion imaging	Web (n=45)	17 (37.7)	8 (17.7)	7 (15.5)	6 (13.3)
	Video (n=12)	3 (25.0)	1 (8.3)	1 (8.3)	1 (8.3)
	Twitter (n=23)	2 (8.6)	1 (4.3)	0 (0.0)	1 (4.3)
Choosing Wisely stress test	Web (n=17)	12 (70.5)	0 (0.0)	11 (64.7)	13 (76.4)
	Video (n=14)	5 (33.3)	2 (14.3)	6 (42.9)	7 (50.0)
	Twitter (n=14)	1 (7.1)	1 (7.1)	3 (21.4)	9 (64.3)

## Discussion

### Principal Findings

In this pilot sample of Internet and social media content regarding nuclear MPI, our search for content related to Choosing Wisely was significantly more likely to discuss appropriateness of testing, accuracy of MPI, and radiation. In fact, the topic of test appropriateness was only discussed in 4% of content found with non–Choosing Wisely searches. This finding is disappointing given that Appropriate Use Criteria (AUC) for nuclear MPI were first published in 2005 [6]. From that time until the most recent update of the AUC in 2013, there appears to have been no appreciable decrease in the rate of inappropriate MPI in the published literature [4,7]. Similar to the lack of Internet content related to appropriateness, physician and provider awareness of appropriateness is low. In a recent survey, 36.6% of respondents had never heard of AUC and only 12.5% reported using them regularly [8].

We were reassured when we did not observe any content that actively encouraged inappropriate MPI (asymptomatic screening, low risk patient screening, or annual testing as part of a cardiology evaluation). This would suggest that publicly searchable information on the Internet is not a significant contributor to the unnecessary use of this particular testing modality.

### Limitations

This investigation has limitations including a small sample size and limited search resources. A more robust methodology may include direct observation or mixed methods assessment of Internet search and social media users for greater detail of their opinions and understanding of unnecessary testing.

### Conclusions

Our findings add to a growing body of literature examining the interface between the medical community and the Internet or social media [9,10]. An investigation which took a similar approach to ours and focused on myocardial infarction also found both inconsistency in the content and lack of substance for relevant concepts such as prevention and risk factors [11]. These authors and others have called for more authoritative content to be developed for these platforms which patients are using to gather information and make decisions about care [12]. Development of such authoritative content may be an important role for the future of the American Board of Internal Medicine Foundation and its partners in the Choosing Wisely campaign. Specific consideration should be given to the format, audience needs, and ideal vehicles for distribution when new content is developed.

## Abbreviations

AUC: appropriate use criteria
MPI: myocardial perfusion imaging
OR: odds ratio
